# Complete mitochondrial genome of oriental pratincole *Glareola maldivarum* (Charadriiformes: Glareolidae) and its phylogenetic relationship

**DOI:** 10.1080/23802359.2022.2098070

**Published:** 2022-07-22

**Authors:** Caichun Peng, Xue Gou, Shize Li, Cheng Wang, Lang Mu, Shuhan Yang, Canshi Hu, Mingming Zhang, Haijun Su

**Affiliations:** a Forestry College, Guizhou University, Guiyang, China; b Research Center for Biodiversity and Natural Conservation, Guizhou University, Guiyang, China; c College of Life Sciences, Guizhou University, Guiyang, China; d Department of Food Science and Engineering, Moutai Institute, Renhuai, China

**Keywords:** Complete mitogenome, oriental pratincole, *Glareola maldivarum*, mitochondrial DNA

## Abstract

Oriental Pratincole *Glareola maldivarum* is a widely distributed water bird species of the Charadriiformes group across Eurasia. In this study, we reveal the mitochondrial genome sequence of the Oriental Pratincole for the first time. The length of the mitochondrial genome is 18,422 bp and contains 2 ribosomal RNA genes, 13 protein coding genes, 22 transfer RNA genes, and 2 D-loop sequences. We further provide a phylogenetic tree showing relationships among Oriental Pratincole and other Charadriiformes species.

The Oriental Pratincole (*Glareola maldivarum* Forster, JR, 1795). Is a common and small waterbird species of the genus *Glareola*, with a body length between 20 and 28 cm and a weight between 53 and 101 grams (Zhao 2001). The species has a short black bill, a dark brown upper body, a white waist and abdomen, and a dark brown fork-shaped tail (Nie 2018). Species mainly inhabits lakes, river and swamps of open plains (Liu and Chen 2021). The Oriental Pratincole is distributed across Eurasia, SE Asia, the Indian subcontinent, Pacific islands, and most areas of China (Brazil 2009). In China, Oriental Pratincole was recorded in all provinces except Xinjiang, and Guizhou (Zheng 2017). Authors, however, observed this species and collected the samples in Guizhou province on October 22, 2020.

In this study, the authors collected the specimen of the species from a pond in Huaxi Country, Guizhou Province China (N26.399519, E106.574858) on October 22, 2020, that died accidentally. The collected sample was stored and preserved separately in 99% ethanol before fixation in the Research Center for Biodiversity and Natural Conservation, Guizhou University (voucher number GZUNZ20210206001, contact person: Caichun Peng; email: pcc1582479121@139.com). The study has been approved by the Guizhou University Subcommittee of Experimental Animal Ethics, No.EAE-GZU-2020-P023. The extraction was performed using DNA Rapid Extraction Kit (Beijing Aidlab Biotechnologies Co., Ltd). The mitochondrial genomes of *G. pratincola* (MN356161.1) were used to design primers for polymerase chain reaction (PCR) and used as the template for gene annotation. Mitogenome was sequenced on an ABI 3730 using the Sanger sequencing protocol. The sequenced genome of *G. maldivarum* was submitted to the NCBI database (GenBank number: BankIt2427067 MW579776) and examined for its phylogenetic relationship with other select Charadriiformes species.

According to the results, complete mtDNA sequences of the Oriental Pratincole were 18,422 bp. The overall base composition was: A, 31.78%; C, 31.52%; G, 12.96% and T, 23.75%. The content of A + T is 55.53%, which is within the range for avian mitogenomes (51.6-55.7%; Haring et al. 2001). It has a typical circular mitochondrial genome containing 13 protein-coding genes, 22 transfer RNAs, 2 ribosomal RNAs and 2 non-coding A + T-rich regions, which are usually found in birds (Boore 1999). Of the 13 protein-coding genes, 9 utilize the standard mitochondrial start codon ATG. However, COI and ND5 use GTG, ND3 use ATA, and ND6 use CTA as initiation codons. TAA is the most frequent stop codon, although COI end with AGG, ND5 end with AGA, ND6 end with CAT, and COIII and ND4 stop with the single nucleotide T. The 12S rRNA is 973 bp, and the 16S rRNA is 1593 bp in length. They are located between tRNA-Phe and tRNA-Leu, separated by tRNA-Val. All tRNAs possess the classic cloverleaf secondary structure, as observed in other bird mitogenomes (Bernt et al. 2013).

The authors used MEGA6 (Tamura et al. 2013) to construct a phylogenetic tree using the maximum-likelihood method (Figure 1). Based on mitogenome sequences of *G. maldivarum* gained and other twelve Charadriiformes species (two of them belong to the same genus as *G. maldivarum*) as also reported by Gou et al. (2021). In the ML tree, *G. maldivarum* was clustered as an independent clade with the boot support value of 100, even with the two relative species of *Glareola* (Figure 1). The complete mitotic genome of *G. maldivarum* is conducive to the protection of non-flagship species and provides basic genetic data for the evolutionary research of charadriformes.

**Figure 1.**
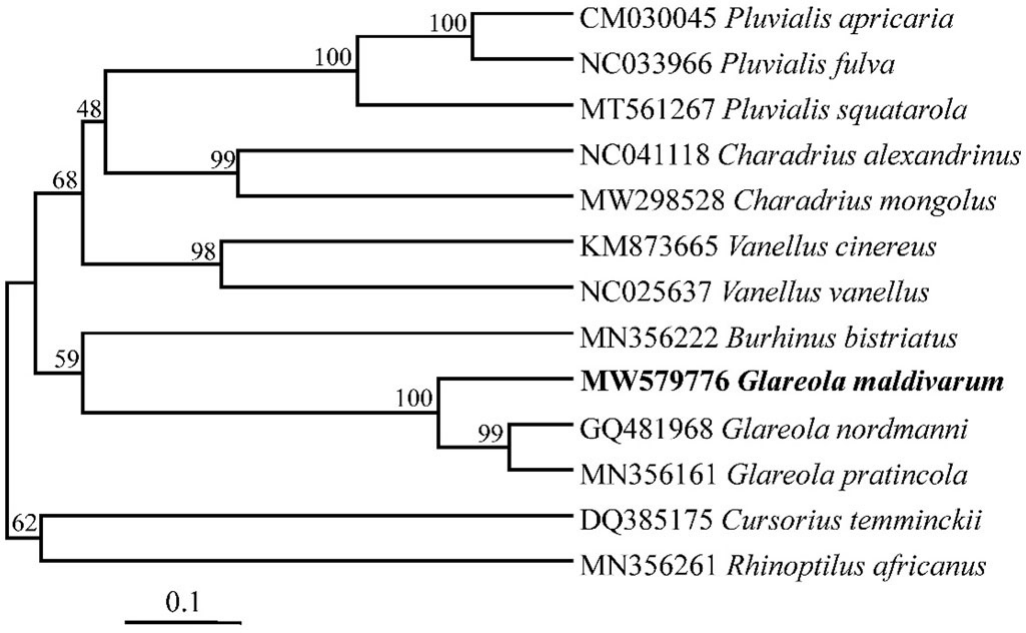
Maximum likelihood tree based on mitogenome sequences of three *Glareola* species and ten Charadriiformes. Number nodes are bootstrap supports.

## Data Availability

The genome sequence data that support the findings of this study are openly available in GenBank of NCBI at [https://www.ncbi.nlm.nih.gov] under the accession no. BankIt2427067 MW579776. The associated BioProject, and Bio-Sample numbers are PRJNA741338, GZUNZ20210206001 respectively.
